# Molecular Mechanisms Underlying Apple Extract Ameliorates Depression‐Incident Cognitive Dysfunction Based on Network Pharmacology

**DOI:** 10.1002/fsn3.70408

**Published:** 2025-06-08

**Authors:** Yue Chen, Shunqiang Yang, Huihuang Shi, Jizhe Cui, Zhu Li

**Affiliations:** ^1^ College of Agronomy and Life Sciences Zhaotong University Zhaotong China

**Keywords:** apple extract, cAMP, CUMS, inflammatory response, network pharmacology

## Abstract

The apple was found to be involved in antidepressant properties. Thus, this study aimed to explore the effects of apple extract (AE) on depression and to elucidate the underlying mechanisms. Our network pharmacology analysis indicated that AE may exert its antidepressant effects through the regulation of inflammatory responses and the cAMP signaling pathway. Behavioral tests showed that AE significantly improved spatial learning, exploratory behavior, anhedonia, and despair in CUMS mice. Biochemical analyses revealed that AE increased synaptic density in the hippocampus, enhanced brain levels of 5‐hydroxytryptamine (5‐HT) and gamma‐aminobutyric acid (GABA), and reduced the expression of pro‐inflammatory cytokines TNF‐α, IL‐1β, and IL‐6 in both serum and brain tissue. In vitro, AE demonstrated significant neuroprotective effects against CORT‐induced cytotoxicity. Furthermore, AE treatment upregulated the levels of cAMP, phosphorylated PKA (pPKA), and phosphorylated CREB (pCREB) while downregulating PTGS2 expression, suggesting that the cAMP/PKA/CREB/PTGS2 signaling pathway is a key mechanism underlying the antidepressant effects of AE. Therefore, our study indicated that AE can improve cognitive dysfunction and alleviate depressive‐like behavior by targeting the cAMP/PKA/CREB/PTGS2 signaling pathway in CUMS mice. This study paved the way for the clinical application of AE as a novel treatment for depression and provided new targeted therapeutic strategies.

## Graphical abstract

This study aimed to investigate the effects of apple extract (AE) on depression and its underlying mechanisms, revealing that AE may exert antidepressant effects by regulating inflammatory responses and the cAMP signaling pathway. Behavioral tests and biochemical analyses demonstrated that AE significantly improved depressive‐like behaviors and cognitive dysfunction in CUMS mice by increasing synaptic density in the hippocampus, enhancing brain levels of 5‐hydroxytryptamine and gamma‐aminobutyric acid, and reducing pro‐inflammatory cytokines. The findings indicate that the cAMP/PKA/CREB/PTGS2 signaling pathway plays a crucial role in the antidepressant effects of AE, paving the way for its potential clinical application as a novel treatment for depression.

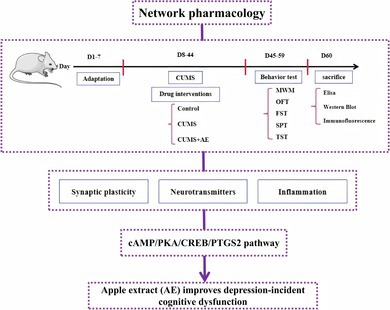

## 1 Introduction

Depression is a mental disorder characterized by high prevalence, high relapse rates, and elevated suicide risk. Clinically, it manifests as marked and sustained emotional disturbances, diminished appetite, anhedonia (loss of interest or pleasure in most activities), cognitive slowing, and social withdrawal. In severe cases, patients may exhibit hallucinations and are at risk of self‐injurious behaviors or suicide, posing a substantial threat to public health (Kim et al. 2024; Yuan et al. 2020). Currently, widely accepted theories regarding the pathophysiology of depression encompass the neurotransmitter hypothesis, the hypothalamic–pituitary–adrenal (HPA) axis hypothesis, the inflammatory hypothesis, the neuroplasticity hypothesis, and the epigenetic hypothesis, among others. However, these hypotheses do not fully elucidate the complex pathophysiological mechanisms underlying depressive disorders (Cui et al. 2024). It is estimated that by 2030, depression will become the leading cause of the global disease burden, thereby imposing significant psychological stress and economic burdens on families and society (Malhi and Mann 2018). Depression exhibits a high degree of heterogeneity, with its etiology and pathophysiological mechanisms being exceedingly complex. This complexity makes it challenging to elucidate the pharmacological mechanisms of antidepressant treatments, which severely limits the development and application of effective medications. Therefore, researching the mechanisms of antidepressant action, developing new antidepressant drugs, and reducing the incidence of depression hold significant importance for both societal well‐being and economic development.

Currently, the treatment of depression primarily relies on pharmacotherapy, and a variety of antidepressant medications have been developed. However, pharmacological treatments often come with significant limitations, including strong dependency, notable side effects, and in some cases, treatment‐resistant depression (Carvalho et al. 2016; Duman and Aghajanian 2012). Therefore, the search for and development of complementary and alternative medicines that can prevent or treat depression is of great significance for improving the health of patients with depression. Traditional Chinese Medicine (TCM) herbs, due to their multi‐component, multi‐target, multi‐signal pathway, and multi‐effect characteristics, along with relatively low toxicity and side effects, align well with the multifactorial, multi‐mechanistic, and multi‐theoretical nature of depression. This makes TCM, especially the food products with both medicinal and edible properties as complementary and alternative therapies, a focal point in the research of antidepressant therapies. Among these, the apple, which is the fruit of a deciduous tree belonging to the Rosaceae family, originally native to Europe and central Asia, is commonly cultivated in Yunnan, Sichuan, Tibet, Liaoning, and Gansu provinces in China, and is widely enjoyed by people. The city of Zhaotong in Yunnan Province boasts unique natural resources and climatic conditions, producing apple with distinctive qualities characterized as being “early, sweet, fragrant, crisp, and colorful.” After nearly a century of introduction, cultivation, and development, Zhaotong has become the largest high‐quality apple production base in southern China. In ancient Chinese medical literature, such as “Ming Yi Bie Lu” (Famous Physicians' Additional Records), the apple was recorded as having medicinal value. As a fruit with both medicinal and dietary uses, the apple possesses high nutritional and economic value. It contains a large amount of bioactive substances, including flavonoids, phenolic acids, and triterpenes. The main flavonoids found in the apple include flavones, flavonols, flavanols, anthocyanins, and chalcones. To date, 35 types of flavonoids have been identified in the apple, such as catechin, epicatechin, and quercetin. Additionally, 21 types of phenolic acids, including p‐coumaroylquinic acid, quinic acid, chlorogenic acid, and caffeic acid, and 24 types of triterpenes, such as ursolic acid and β‐sitosterol, have been discovered (Lee et al. 2003; Schieber et al. 2001; Wang, Wang, Liu, et al. 2024). Due to its rich content of various bioactive components, the apple exhibits multiple pharmacological activities. Research has shown that the apple extract possesses a range of biological functional properties, including the ability to scavenge free radicals, anti‐inflammatory effects, antioxidant activity, inhibition of tumor cell proliferation, and anti‐aging properties (He and Liu 2008; Li et al. 2019; Luo et al. 2016; Xiang et al. 2011; Zielinska et al. 2019). Indeed, the apple contains a variety of bioactive components that play important roles in the prevention of chronic diseases and in promoting overall health. However, there is limited reporting specifically on the antidepressant effects of apple. In TCM, depression falls under the category of *Yuzheng*, with the primary pathological location being the liver. According to the *Suwen*, one of the foundational texts of TCM, “anger injures the liver.” The liver is responsible for storing blood and regulating the smooth flow of *Qi*. When the liver functions properly, *Qi* and blood circulate harmoniously, leading to a balanced and cheerful mood. Conversely, dysfunction of the liver can lead to abnormal emotional states. Clinical observations and anecdotal evidence suggested that the apple may have liver‐nourishing and detoxifying properties. Furthermore, it has been noted that when patients with depression inhale the apple aroma, they experience a reduction in feelings of oppression, an improvement in mood, and a relief from insomnia symptoms. These observations indicated that the apple may possess potential antidepressant properties. To summarize, while the apple is well‐known for its general health benefits and its role in preventing chronic diseases, emerging evidence suggests that it may also have specific benefits for mental health, particularly in the context of depression. The liver‐nourishing and mood‐enhancing effects of apple, as observed in TCM, provide a promising area for further research into their potential as a complementary and alternative therapy for depression (“Health Benefits of Apples,” 2013). An increasing body of evidence supports the antidepressant effects of individual components found in apple, such as chlorogenic acid and epicatechin, in mouse models of depression (Martínez‐Damas et al. 2021; Song et al. 2019). Moreover, proanthocyanidin B2 has been demonstrated to decrease blood–brain barrier (BBB) leakage and enhance the diminished expression of the tight junction protein zonula occludens‐1 (ZO‐1) in the cortex and striatum, thus reinforcing the BBB. This suggests that proanthocyanidin B2 may also have antidepressant effects (Wu et al. 2015). Recent studies have demonstrated that apple polyphenol extracts improve depressive‐like behaviors in mice fed a high‐sugar diet by inhibiting inflammation in the gut‐brain axis (Xie, Wu, et al. 2024). Another study indicated that apple polyphenol extracts alleviate depressive‐like behaviors in high‐sugar diet‐fed mice through the farnesoid X receptor (FXR)‐mediated hepatoenteric‐brain axis bile acid circulation (Xie, Shang, et al. 2024). However, these studies have focused on the antidepressant effects of specific components in the apple, which may not fully reflect the interactions and synergistic effects of the multiple bioactive compounds present in the apple. Therefore, investigating the impact of apple extract mixtures on depression could provide new insights that might not be achievable with individual components alone. This holistic approach could help in understanding the comprehensive therapeutic potential of apple in the context of depression and may lead to the development of more effective, natural‐based treatments.

Network pharmacology integrates systems biology, polypharmacology, and computational analysis techniques to construct a “drug‐component‐target‐disease” interaction network. This approach reduces the blindness of research and enhances research efficiency. By building multi‐level networks, it systematically elucidates the mechanisms of drug actions at the gene, molecular, and cellular levels, laying the foundation for the development of new drugs (Zhang et al. 2023). PharmMapper is a tool that integrates databases such as DrugBank, BindingDB, Target Bank, and PPTD to perform reverse pharmacophore modeling for target prediction. It can rapidly predict the targets of drug molecules (Wang et al. 2017). Computer‐Aided Drug Design (CADD) is a method that utilizes computer technology and simulation software to design, screen, and optimize drug molecules based on the structure and function of their targets. CADD is a crucial technique in modern drug discovery and development. This study initially aimed to establish a predictive model for the antidepressant activity of apple using network pharmacology, to predict the potential targets and mechanisms of action of the antidepressant components in the apple, thereby providing a theoretical basis for further research.

Therefore, building upon previous research, this study will integrate network pharmacology with traditional pharmacodynamic techniques and high‐throughput sequencing technologies to systematically elucidate the antidepressant mechanisms of apple from Zhaotong, Yunnan. This comprehensive approach aims to provide a scientific basis for the safe and extensive clinical application of apple and to offer a novel perspective for the development and utilization of apple in the Zhaotong region of Yunnan.

## 2 Materials and Methods

### 2.1 Screening of Active Ingredients of Apple and Related Targets

We conducted a literature review using the CNKI and PubMed databases to gather information on the active components of apple. The canonical SMILES of the relevant compounds were input into the Traditional Chinese Medicine Integrated Database (TCMID), ECTM, and Swiss ADME (http://www.swissadme.ch/) to select compounds with high gastrointestinal absorption and favorable drug‐like properties as the target compounds. Subsequently, the canonical SMILES of these target compounds were input into the Swiss Target Prediction database (http://www.swisstargetprediction.ch/), with the species set to “ *Homo sapiens* .” Targets with a probability greater than 0.7 were selected as the potential targets of the active components in apple.

### 2.2 Screening of Depression‐Related Targets

Depression targets were retrieved from GeneCards, DrugBank, Therapeutic Target Database, OMIM and DisGeNET databases using the keyword “Depression”. The species was restricted to “ *Homo sapiens* ”. The duplicate targets were removed, and the names were standardized using the UniProt database.

### 2.3 Acquisition of Apple‐Depression Overlapping Targets

Targets related to apples and those associated with depression were input into Venny2.1.0 to create an overlapping target set for further analysis.

### 2.4 Establishment of Protein–Protein Interaction (PPI) and Screening of Core Active Targets

The overlapping targets obtained were uploaded to the STRING database, specifying “ *Homo sapiens* ” as the protein species and setting the minimum required interaction score to the highest confidence level (0.900). Isolated nodes were removed, and the online tools were used to predict the interactions among the genes. The results were visualized as a Protein–Protein Interaction (PPI) network for the antidepressant activity of apple using Cytoscape 3.9.1 software. Topological analysis of the PPI network was carried out using the CentiScape 2.2 plugin within Cytoscape 3.9.1. The size and color intensity of the nodes represent the Degree values, and the thickness of the edges reflects the Combined Score. The top 6 nodes ranked by Degree value were selected as the core target network, and the core active targets were identified.

### 2.5 GO and KEGG Pathway Enrichment Analyses

The targets overlapping between apple‐related and depression‐related genes were uploaded to the Metascape database. GO enrichment analysis and KEGG pathway enrichment analysis were then conducted on these intersecting targets.

### 2.6 Preparation of Apple Extract

The apples ( *Malus pumila* Mill. cv. Ruixue) were harvested in Zhaotong City, Yunnan Province, China. The apples underwent thorough washing with water, were shade‐dried to remove surface moisture, and subsequently ground into a fine powder. A quantity of this dried powder (50 g) was subjected to extraction using 70% ethanol (5 L) for a duration of 12 h. After the extraction process, the resulting liquid was concentrated using a rotary evaporator over a period of 2 h. To ensure complete removal of ethanol, the concentrated extract was diluted with water and subjected to another round of concentration. Following this, the processed liquid was carefully filtered through filter paper to remove any residual particulates. The filtrate was then placed on a freezing tray where it remained frozen for 24 h. Subsequently, the frozen material underwent freeze‐drying for an extended period of 36 h, ultimately yielding a completely dried apple extract weighing 10 g. This final product was then dissolved in a solvent system (10% DMSO + 40% PEG300 + 5% Tween‐80 + 45% Saline) and then diluted 2‐fold with saline for experimental use.

### 2.7 Chemical Composition Analysis

The composition of 70% ethanol extracts from the “Ruixue” variety of *Malus pumila* Mill. apples was quantitatively evaluated via High‐Performance Liquid Chromatography (HPLC). An HPLC setup from Shimadzu (Kyoto, Japan) was employed. The data collection process relied on the Shimadzu Lab Solution software. This separation occurred at room temperature, with detection occurring at a wavelength of 273 nm. The mobile phase was composed of water containing 0.1% trifluoroacetic acid (designated as A) and acetonitrile (designated as B), and gradient elution was applied in the following manner: within 0–15 min, the proportion of B was 5%–10%; from 15–30 min, it was 10%–30%; during 30–50 min, 30%–50%; in 50–70 min, 50%–80%; from 70–75 min, 80%–100%; between 75–85 min, it remained at 100%; from 85–95 min, it decreased from 100% to 5%; and from 95–100 min, it stayed at 5%. The flow rate was set at 1 mL per minute, and the temperature was maintained at 37°C. The main compounds identified in the 70% ethanol extract of “Ruixue” apples were determined. These major compounds were epicatechin (14.50% ± 1.27%), caffeic acid (3.40% ± 0.35%), gallic acid (2.60% ± 0.28%), phloretin (2.95% ± 0.25%), and quercetin (0.90% ± 0.08%).

### 2.8 Animals

Thirty 8‐week‐old male C57BL/6J mice, each weighing around 23 ± 2 g, were sourced from the Yunnan University Experimental Animal Centre (approval number SYXK (Dian) 2021–0002). These mice were housed in groups of 5 to 6 per cage under controlled environmental conditions: a temperature of 22°C ± 2°C, relative humidity of 55%, and a 12‐h light/dark cycle. Food and water were provided ad libitum throughout the study, except during specific experimental procedures. All procedures were reviewed and approved by the Zhaotong University Animal Ethics Committee (approval number 20220003A8), ensuring compliance with stringent ethical standards. These procedures were carried out following the guidelines set forth by the National Institutes of Health (NIH) Guide for the Care and Use of Laboratory Animals, which are internationally recognized for promoting the humane treatment of animals in research, and all personnel involved held valid certifications for working with laboratory animals.

### 2.9 Animal Grouping and Drug Administration

Before the experiment, the animals were subjected to a one‐week acclimation period.

Following this, the male C57BL/6J mice were randomly allocated into three distinct groups: normal control group, CUMS group, and CUMS + AE (administered at a dose of 500 mg/kg) group. All mice received physiological saline daily for three days before the experiment to familiarize them with oral gavage (i.g.). The apple extract was administered via intragastric gavage at a constant volume and given 30 min before the initiation of the CUMS protocol. Throughout the 5‐week experimental period, these treatments were administered daily.

### 2.10 CUMS Procedure

The CUMS paradigm was conducted as previously described with modifications (Nollet et al. 2019). Briefly, all groups, excluding the control group, were exposed to a total of 13 chronic stressors. These included tail pinch (2 min), shaking the mouse cage (15 min), exposure to a novel object (24 h), swimming in 4°C ice water (5 min), physical restraint (3 h), tilting the cage at a 45° angle (24 h), olfactory irritation (24 h), food deprivation (24 h), exposure to strobe lighting (5 h), reversal of day and night cycles (24 h), moist bedding (24 h), water deprivation (24 h), and mixed housing conditions (24 h). Stressors were randomly assigned over a period of 7 weeks, with one or two unpredictable stimuli given to the animals each day. Care was taken to ensure that no stressor was repeated on consecutive days, preventing the animals from anticipating the stimuli.

### 2.11 Behavioral Tests

Behavioral assessments were performed following 5 weeks of CUMS exposure by an evaluator who was unaware of the treatment groups.

#### 2.11.1 Morris Water Maze (MWM)

The Morris Water Maze (MWM) was used to assess the spatial cognitive function of the mice as described in our previous study (Li et al. 2020). Briefly, the experimental setup included a circular stainless steel pool with a diameter of 120 cm and a height of 40 cm. The pool's interior was painted black and divided into four equal quadrants. It was filled with water to a depth of 30 cm, which was kept at a temperature of 22°C ± 2°C. A circular escape platform, measuring 10 cm in diameter, was positioned in one of the quadrants designated as the target quadrant, submerged approximately 1.5 cm beneath the water surface. The behavioral test was structured into two main phases: an acquisition training phase followed by a spatial probe test. During the acquisition training, subjects were required to locate the hidden platform using visual cues surrounding the pool. This phase helped assess their ability to learn and remember the platform's location over multiple trials. In the subsequent spatial probe test, the platform was removed, and the animals' search patterns were analyzed to evaluate their retention and recall of the platform's previous position. Throughout both phases, detailed data such as the swim path, swim distance, and swim time were meticulously recorded using a high‐resolution camera system coupled with automated tracking and analysis software.

#### 2.11.2 Open Field Test (OFT)

The Open Field Test (OFT) was often used to reflect the curiosity, exploration, and locomotion of mice in a new environment, and the experimental procedure was as described previously (Wang et al. 2019). Briefly, the test system comprised an open‐field apparatus with dimensions of 50 × 50 × 50 cm, monitored by a digital camera positioned directly above its center. Each mouse was placed individually at the center of the apparatus and given the opportunity to explore freely for a total duration of 6 min. Following an initial 2‐min acclimation period, the number of crossings was recorded during the subsequent 4 min. To ensure cleanliness and prevent any olfactory cues from previous trials, the apparatus was thoroughly cleaned with 75% alcohol between each trial, removing excrement and odors.

#### 2.11.3 Sucrose Preference Test (SPT)

The Sucrose Preference Test (SPT) was utilized to evaluate anhedonia, or the loss of pleasure in mice. The SPT was conducted as previously described, with minor modifications (Liu et al. 2018). Briefly, prior to the test, the mice underwent a 24‐h acclimation period to a 1% sucrose solution (w/v). Following acclimation, the mice were subjected to a 12‐h period of water and food deprivation. Subsequently, each mouse was individually housed and given access to two bottles: one containing tap water and the other 1% sucrose solution, for a duration of 12 h, with the positions of the bottles switched after 6 h to prevent positional bias. After the 12‐h period, the water bottle was removed, and the consumption volumes of both the sucrose solution and plain water were recorded. Sucrose preference, which represents anhedonia, was then calculated using the formula: sucrose preference index (%) = sucrose solution consumption/total fluid intake × 100%.

#### 2.11.4 Forced Swim Test (FST)

The Forced Swim Test (FST) was conducted as previously described (Duman et al. 2007; Porsolt et al. 1977). Briefly, each mouse was individually introduced into a cylindrical water tank measuring 30 cm in height and 40 cm in diameter. The water temperature was maintained at 22°C ± 2°C to ensure consistent experimental conditions. Mice were allowed to remain in the tank for a total duration of 6 min. During this period, immobility time was recorded specifically during the final 4 min. Immobility was defined as the mouse positioning its body perpendicular to the water surface, with only its nose above water, or when the mouse floated almost motionless on the water's surface. This behavioral assessment aimed to evaluate the level of despair or depressive‐like behavior in the mice. By focusing on the last 4 min, the test minimized the impact of initial stress reactions and provided a more accurate measure of sustained immobility, which is indicative of reduced escape‐related behaviors.

#### 2.11.5 Tail Suspension Test (TST)

The Tail Suspension Test (TST) was performed to evaluate behavioral despair of mice as previously described, with minor modifications (Steru et al. 1985). Briefly, each mouse was suspended approximately 20 cm above the ground, with the rope positioned about 1 cm from the tip of the tail. The test duration was 6 min. During this period, any mouse that remained completely motionless was considered immobile. The total immobility time was specifically recorded during the last 4 min of the observation to assess behavioral despair. To elaborate further, this method aimed to evaluate the level of despair in mice by focusing on their immobility behavior. By excluding the initial 2 min and recording only the final 4 min, the test minimized the influence of initial stress reactions and provided a more accurate measure of sustained immobility.

### 2.12 Tissue Sample Collection

After behavioral tests, the mice were deeply anesthetized by CO_2_. On day 60, different sample materials were collected from each group of mice. Blood was first drawn from the mice's eyes using a capillary tube and transferred to EP tubes, where it was allowed to clot at room temperature for 1 h. The samples were then centrifuged at 4000 rpm for 15 min at 4°C. The resulting serum was carefully aspirated and stored at −20°C for further analysis. Simultaneously, brain tissues were rapidly harvested and snap‐frozen in liquid nitrogen before being stored at −80°C. For histological analysis, mice were deeply anesthetized using CO_2_ and then transcardially perfused with 20 mL of pre‐cooled phosphate‐buffered saline (1× PBS, pH 7.4). Brain tissues intended for immunofluorescence assays were extracted and post‐fixed overnight in the same PBS solution. These tissues were subsequently dehydrated through a graded series of sucrose solutions at 4°C to prepare them for sectioning and staining. The isolated tissues were then embedded in optimal cutting temperature (OCT) compound.

### 2.13 Immunofluorescence Assay

The immunofluorescence assay was conducted according to the protocol previously described by us, with minor modifications (Li et al. 2024). Briefly, brain tissues were slowly frozen and then sectioned into serial coronal slices, each 20 μm thick. The sections were rinsed three times to remove any debris and permeabilized with 0.1% Triton X‐100 for 20 min to allow better penetration of antibodies. Following this, the sections were incubated in a blocking solution containing 1.5% fetal bovine serum for 1 h at room temperature to minimize non‐specific binding. Subsequently, the sections were incubated overnight at 4°C with primary antibodies: PSD‐95 (1:500, ab18258, Abcam) and Synaptophysin (1:400, ab8049, Abcam). After another round of PBS rinsing, the sections were incubated with fluorescent‐conjugated secondary antibodies. The immunofluorescence slides were imaged using a confocal laser scanning microscope (LSM980, Carl Zeiss).

### 2.14 Enzyme‐Linked Immunosorbent Assay (ELISA)

Following the completion of the behavioral tests, the enzyme‐linked immunosorbent assay (ELISA) was performed in accordance with the manufacturer's guidelines. ELISA was used to measure levels of inflammatory mediators, including TNF‐α (Solarbio, SEKM‐0034), IL‐6 (Solarbio, SEKM‐0007) and IL‐1β (Solarbio, SEKM‐0002), PTGS2 (Solarbio, SEKM‐0156) and cAMP (Solarbio, SEKSM‐0017) in brain tissue and serum of mice.

### 2.15 Western Blot

The protein sample lysates or precipitates were denatured in sodium dodecyl sulfate (1 × SDS) sample buffer and subsequently separated by 10% SDS‐polyacrylamide gel electrophoresis (SDS‐PAGE). Following separation, the proteins were transferred onto a polyvinylidene difluoride (PVDF) membrane. The membrane was then incubated with appropriate primary antibodies to detect specific proteins. Anti‐PKA (1:1000, ab32514, Abcam) and anti‐phospho‐PKA (1:1000, ab238951, Abcam), anti‐CREB (1:1000, ab32515, Abcam) and anti‐phospho‐CREB (1:1000, ab32096, Abcam) were primary antibodies. Each antibody was diluted according to the manufacturer's recommendations and applied to the membrane to identify the respective proteins of interest. After incubation with the primary antibodies and subsequent washing steps to remove unbound antibodies, the membrane was incubated with the HRP‐labeled secondary antibody (1:4000, A0545, Sigma). Protein visualization was achieved using enhanced chemiluminescence, and their densities were quantified using ImageJ software (NIH).

### 2.16 Cell Culture and Treatments

Neuro‐2a (N2a) cells were cultured in high‐glucose Dulbecco's Modified Eagle Medium (DMEM) supplemented with 10% fetal bovine serum (FBS), 100 U/mL penicillin, and 100 mg/mL streptomycin. The cells were seeded at a density of 1 × 10^5^ cells/mL and maintained at 37°C in a humidified atmosphere containing 5% CO_2_ for 24 h. All cell lines used in our study were confirmed to be mycoplasma‐free through PCR testing. Plated N2a cells were then exposed to corticosterone (CORT) at various concentrations (0, 100, 200, 400, 600, and 800 μmol/L), with a purity of 95%, for 24 h. This treatment was designed to assess the effects of different CORT concentrations on the cells.

### 2.17 Cell Viability Assay

Cell viability was measured using a Cell Counting Kit‐8 (CCK‐8) (Beyotime, C0039). Briefly, inoculate an appropriate amount of cells into 96‐well plates and culture them to allow the cells to adhere to the wall and grow to a suitable state. Perform corresponding drug treatments on cells in different groups. After the cells in each group are intervened with drugs for the corresponding time, discard the original medium in the wells. Add 100 μL of fresh medium to each well, and then add 10 μL of CCK‐8 solution to each well. Place the 96‐well plate back into the cell incubator and continue culturing for 2 h to ensure sufficient reaction between the CCK‐8 solution and the cells. After the culture is completed, use a microplate reader to measure the absorbance values of each group at a wavelength of 450 nm. The experimental results are expressed as the ratios of the absorbance values of other groups to that of the normal group.

### 2.18 Statistical Analysis

Comparisons among the three groups were conducted using one‐way analysis of variance (ANOVA), followed by the Newman–Keuls test for post hoc comparisons. All statistical analyses were performed using SPSS 20.0 and Prism 9.0 (GraphPad Software). For the quantification of immunostaining, assessments were carried out in a blinded manner to minimize bias. Data were expressed as mean ± standard deviation (SD), ensuring clear representation of the central tendency and variability of the measurements. Statistical significance was set at *p* < 0.05, meaning that any *p* values less than 0.05 were considered indicative of statistically significant differences.

## 3 Results

### 3.1 Based on Network Pharmacology, the Potential Targets and Mechanisms of Action for the Antidepressant Effects of AE Have Been Predicted

**FIGURE 1:**
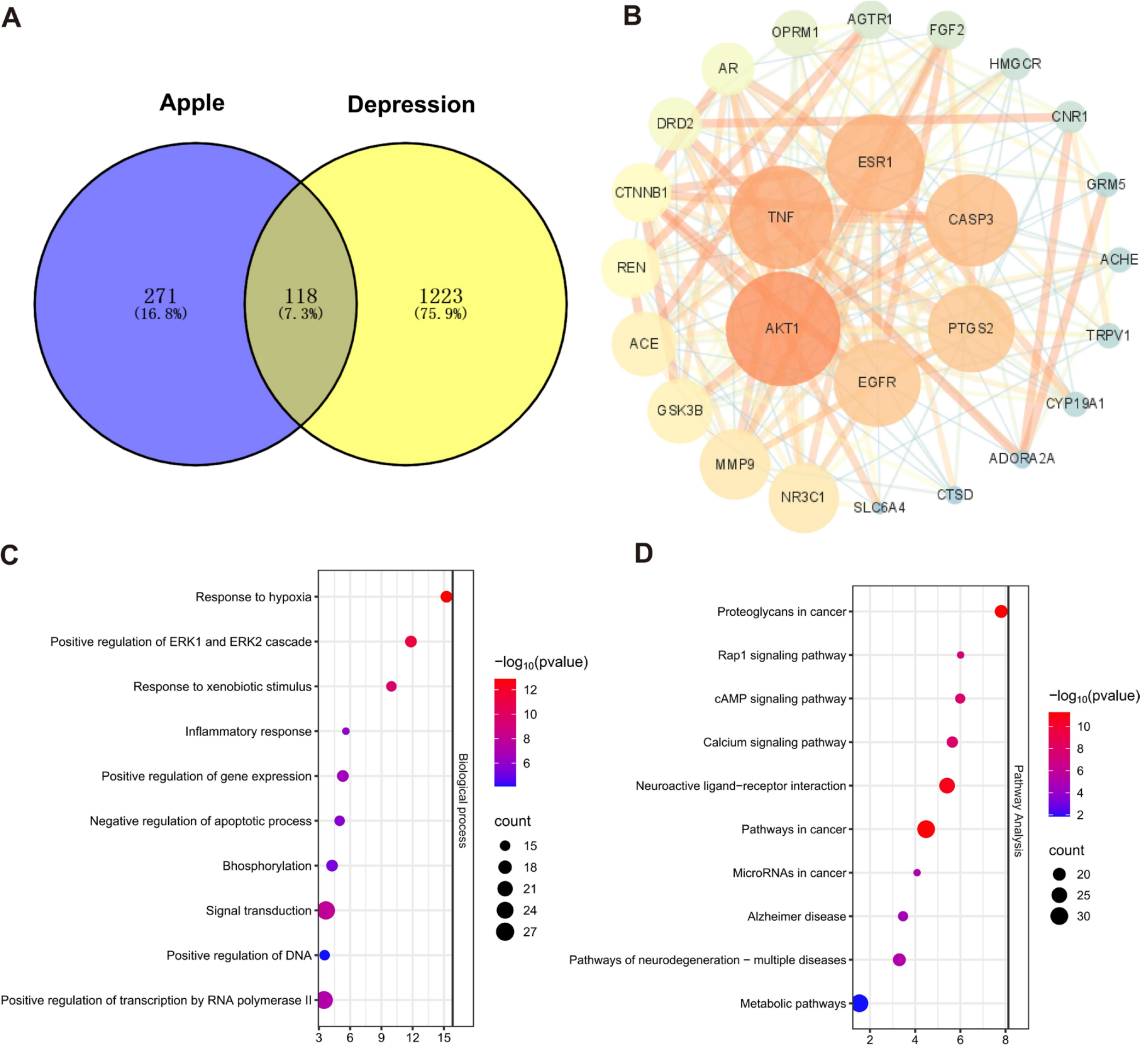
Prediction of potential targets and mechanisms of action for the antidepressant effects of apples. (A) Venn diagram showing the intersection between the targets of apple's active components and depression‐associated disease targets. (B) Protein–Protein Interaction (PPI) network of the intersected targets between the targets of apple's active components and depression‐associated disease targets. (C) Gene Ontology (GO) enrichment analysis. (D) Kyoto Encyclopedia of Genes and Genomes (KEGG) pathway enrichment analysis.

Through the integration of five databases—TCMID, HERB, ETCM, HIT, and TCMIP—along with supplementary literature from CNKI and PubMed, 17 active components of apples were identified, with detailed information listed in Table 1. Using the PharmMapper database and after removing duplicates, standardized gene names were obtained via the UniProt database, resulting in 389 potential targets for the effective components of apple. A total of 1341 depression‐related genes were retrieved from the Genecards database (with a Relevance score ≥ 3). By intersecting the potential targets of apple's active components with depression‐related targets, 118 potential anti‐depressive targets of apple's active components were identified (Figure 1A). Protein–Protein Interaction (PPI) analysis was conducted on these 118 intersecting targets, followed by the selection of the top 6 nodes (AKT1, TNF, ESR1, CASP3, PTGS2, and EGFR) based on Degree value using the CentiScape 2.2 plugin in Cytoscape software, forming the core target network (Figure 1B). To better understand the biological functions and pathways associated with the 118 potential targets, we conducted both Gene Ontology (GO) enrichment analysis and Kyoto Encyclopedia of Genes and Genomes (KEGG) pathway analysis. Together, these complementary analyses provide a comprehensive view of the functional roles and pathway associations of the 118 potential targets. GO analysis revealed that the anti‐depressive effects of apple are mainly enriched in biological processes such as response to hypoxia, response to xenobiotic stimulus, inflammatory response, and positive regulation of gene expression (Figure 1C). KEGG pathway analysis indicated that the key pathways through which apple exerts its anti‐depressive effects include pathways in cancer, cAMP signaling pathway, calcium signaling pathway, and neuroactive ligand‐receptor interaction (Figure 1D). The aforementioned predictive results suggested that the apple may play a crucial role in antidepressant mechanisms, which is associated with inflammatory responses, and the cAMP signaling pathway is likely a key pathway through which apple exerts its antidepressant effects.

**TABLE 1:** Major active components of apples.

Component	CAS number	Canonical SMILES
Quercetin	117‐39‐5	C1=CC(=C(C=C1C2=C(C(=O)C3=C(C=C(C=C3O2)O)O)O)O)O
Phloretin	60‐82‐2	C1=CC(=CC=C1CCC(=O)C2=C(C=C(C=C2O)O)O)O
Taxifolin	480‐18‐2	C1=CC(=C(C=C1C2C(C(=O)C3=C(C=C(C=C3O2)O)O)O)O)O
Anthocyanidin	13306‐05‐3	C1=CC(=C(C=C1C2=[O+]C3=CC(=CC(=C3C=C2O)O)O)O)O
Methyl protocatechuate	2150‐43‐8	COC(=O)C1=CC(=C(C=C1)O)O
Catechin	154‐23‐4	C1C(C(OC2=CC(=CC(=C21)O)O)C3=CC(=C(C=C3)O)O)O
Epicatechin	490‐46‐0	C1C(C(OC2=CC(=CC(=C21)O)O)C3=CC(=C(C=C3)O)O)O
Gallic acid	149‐91‐7	C1=C(C=C(C(=C1O)O)O)C(=O)O
Caffeic acid	331‐39‐5	C1=CC(=C(C=C1C=CC(=O)O)O)O
Ferulic acid	1135‐24‐6	COC1=C(C=CC(=C1)C=CC(=O)O)O
Maleic acid	110‐16‐7	C(=CC(=O)O)C(=O)O
p‐Coumaric acid	4501‐31‐9	C1=CC(=CC=C1C=CC(=O)O)O
Cinnamic acid	140‐10‐3	C1=CC=C(C=C1)C=CC(=O)O
2α‐Hydroxyursolic acid	4547‐24‐4	CC1CCC2(CCC3(C(=CCC4C3(CCC5C4(CC(C(C5(C)C)O)O)C)C)C2C1C)C)C(=O)O
β‐Sitosterol	83‐46‐5	CCC(CCC(C)C1CCC2C1(CCC3C2CC=C4C3(CCC(C4)O)C)C)C(C)C
Ethyl 3,4‐Dihydroxybenzoate	3943‐89‐3	CCOC(=O)C1=CC(=C(C=C1)O)O
2(R)‐Hydroxybutaned‐ioic acid	143435‐96‐5	C(C(C(=O)O)O)C(=O)O

### 3.2 Effect of AE Treatment on Cognitive Functions and Depression‐Like Behaviors in CUMS Mice

**FIGURE 2:**
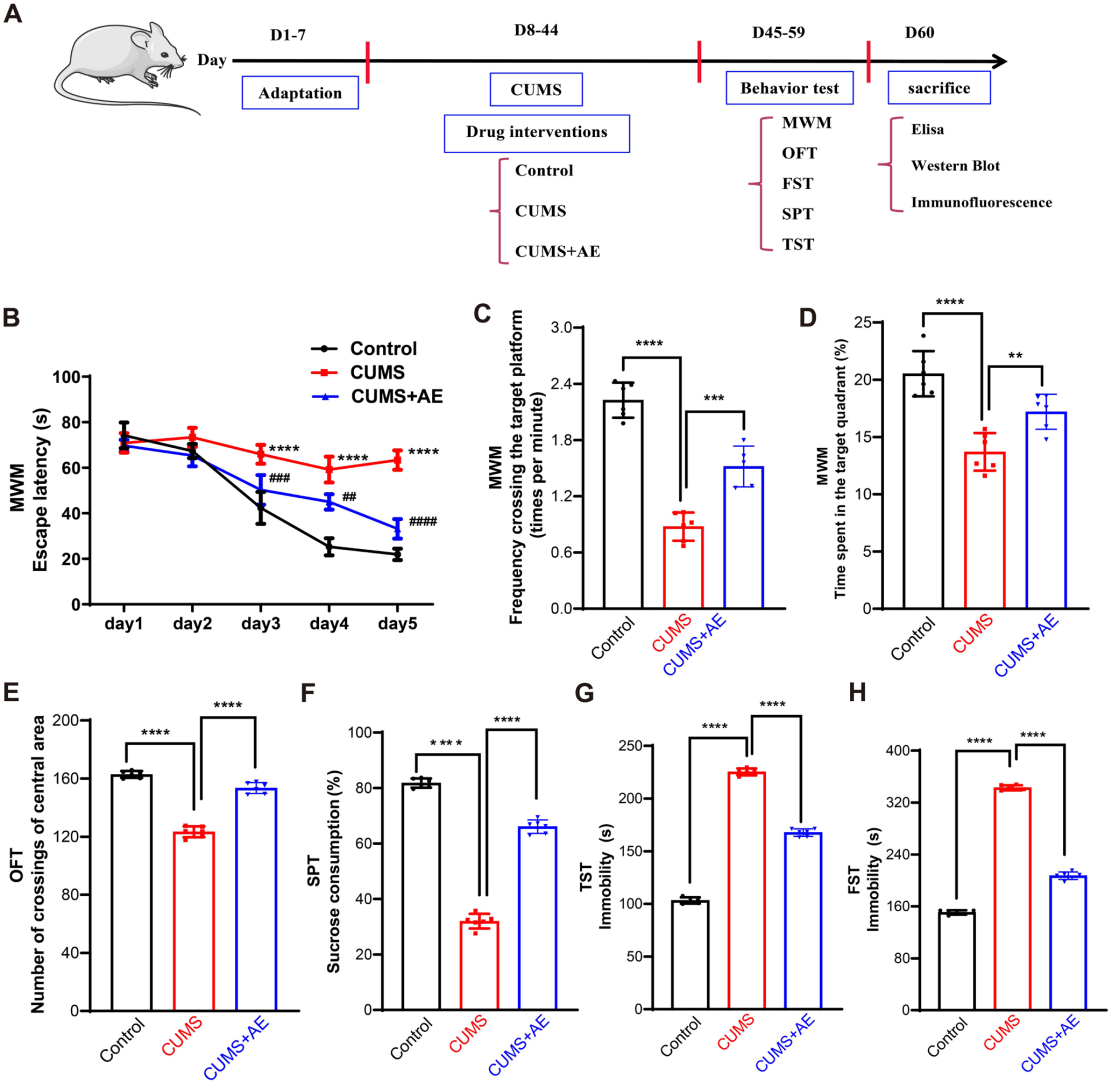
AE treatment ameliorates cognitive dysfunction and depression‐like behaviors in CUMS mice. (A) Diagram of experimental schedule. (B) The latency to the platform of 5‐day acquisition training in MWM. (C) The frequency across the target quadrant in the probe test. (D) The percentage of time spent in the target quadrant in the probe test. (E) Number of crossings of central area in OFT. (F) Percentage of sucrose consumption in SPT. (G‐H) The immobility in the TST (G) and FST (H). All data are presented as the mean ± SD. *n* = 6 mice per group, ^##^ *p* < 0.01, ^###^ *p* < 0.001, and ^####^ *p* < 0.0001 vs. Control group, **p < 0.01, ***p < 0.001, and ****p < 0.0001 vs. CUMS group.

To explore the impact of AE on cognitive function and depression‐like behaviors induced by chronic unpredictable mild stress (CUMS) in mice, we conducted a series of behavioral tests such as MWM, OFT, SPT, TST, and FST. The detailed experimental flow chart is illustrated in Figure 2A. During the acquisition training phase of the MWM, different groups displayed varying levels of spatial learning ability over the 5‐day training period. Treatment with AE significantly decreased the escape latency (Figure 2B). Specifically, the CUMS group showed significantly longer escape latencies compared to the control group on days 3–5, whereas the AE‐treated group exhibited notably shorter escape latencies relative to the CUMS group (Figure 2B). In the probe test, CUMS‐exposed mice crossed the target platform less frequently and spent a significantly lower percentage of time in the target quadrant compared to the control mice. Conversely, the AE group demonstrated a significantly higher frequency of crossing the target platform and a greater percentage of time spent in the target quadrant compared to the CUMS group (Figure 2C,D). In the OFT, CUMS‐exposed mice had a significantly reduced number of grid crossings compared to the control group, while AE administration significantly increased the number of grid crossings compared to the CUMS group (Figure 2E). For the SPT, CUMS‐exposed mice consumed significantly less sucrose than control mice, indicating anhedonia. However, treatment with AE significantly reversed this reduction in sucrose preference and alleviated anhedonia symptoms in CUMS mice (Figure 2F). The desperation state in mice was assessed using the immobility time in both the TST and FST (Figure 2G,H). CUMS stimulation led to a significant increase in immobility time in both tests compared to the control group (Figure 2G,H). Consistent with expectations, AE treatment notably reduced the immobility duration and improved signs of hopelessness in the AE group compared to the CUMS group (Figure 2G,H). Overall, these behavioral tests demonstrated that AE treatment not only enhanced cognitive performance but also produced antidepressant‐like effects in the CUMS‐induced depression model. This comprehensive evaluation underscores the potential therapeutic benefits of AE in mitigating both cognitive deficits and depressive symptoms.

### 3.3 Effect of AE Treatment on Synaptic Plasticity and Neurotransmitter in CUMS Mice

**FIGURE 3:**
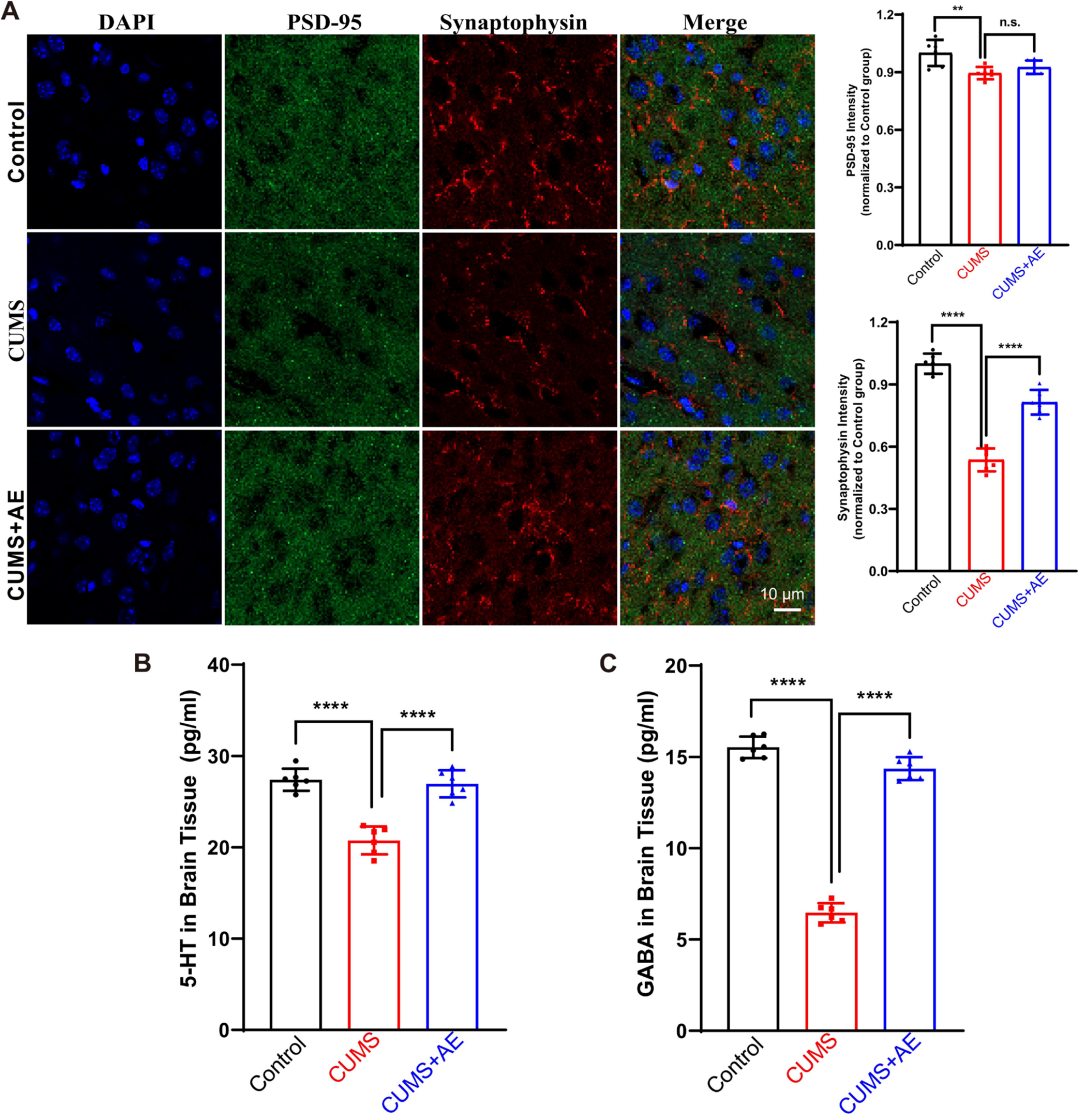
AE plays essential roles in regulating synaptic plasticity and neurotransmitter levels in CUMS mice. (A) Immunofluorescence assay of PSD‐95 and synaptophysin in mice hippocampal tissues. Image analyses and quantification were performed using ImageJ software. (B and C) The levels of neurotransmitters 5‐HT (B) and GABA (C) in mouse brain tissue. All data were presented as the mean ± SD. *n* = 6 mice per group, n.s. > 0.05, **p < 0.01 and ****p < 0.0001 vs. CUMS group.

To determine whether AE influenced synaptic growth in vivo, we performed immunofluorescence assays using hippocampal tissues from mice, focusing on the proteins PSD‐95 and synaptophysin. These markers are crucial for assessing synaptic integrity and density. In the hippocampal tissues of CUMS‐exposed mice, both the relative fluorescence intensities and protein levels of PSD‐95 and synaptophysin were notably lower compared to those in the control group. In AE treatment, the relative fluorescence intensities of synaptophysin significantly increased, but the increase of PSD‐95 was limited, pointing to elevated neuronal synaptic density in vivo (Figure 3A). As the most extensive cohesive neurotransmitter systems in the brain, 5‐hydroxytryptamine (5‐HT, also known as serotonin) and gamma‐aminobutyric acid (GABA) play crucial roles in regulating a wide array of biological functions. These include mood regulation, sleep patterns, appetite control, and energy balance. Therefore, we examined the levels of neurotransmitters 5‐HT and GABA in mouse brain tissue. Compared to the control, the levels of neurotransmitters 5‐HT and GABA were significantly decreased in CUMS mice. The AE intervention led to a significant increase in the levels of 5‐HT (Figure 3B) and GABA (Figure 3C) in brain tissue compared to the CUMS group. Together, these results strongly suggested that AE plays essential roles in promoting synaptic plasticity and balancing neurotransmitter systems.

### 3.4 Effect of AE Treatment on Inflammatory Cytokine in CUMS Mice

**FIGURE 4:**
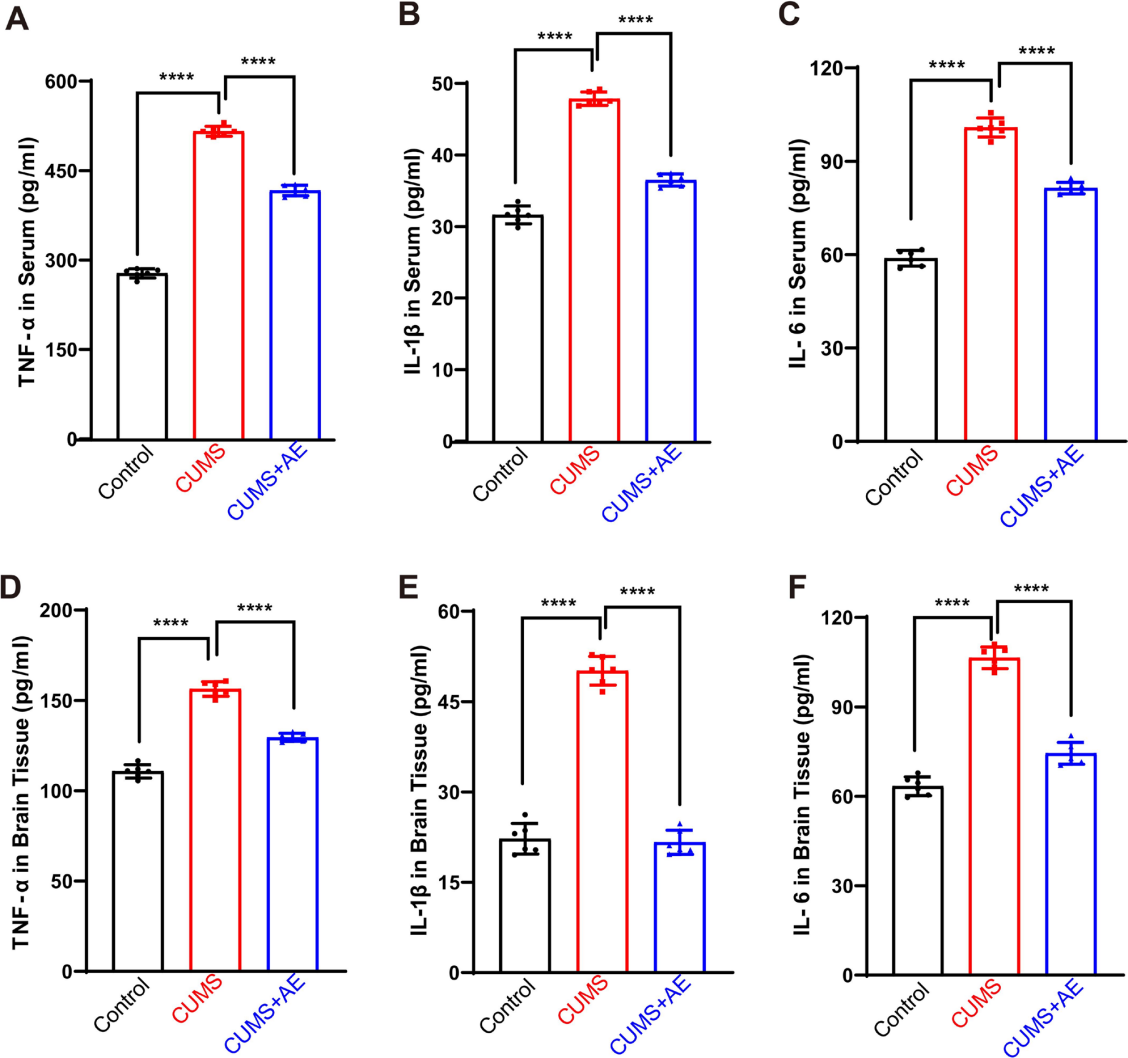
AE inhibited CUMS‐induced inflammatory responses. (A–C) Levels of TNF‐α (A), IL‐1β (B), and IL‐6 (C) in serum. (D–F) Levels of TNF‐α (D), IL‐1β (E), and IL‐6 (F) in brain tissue. All data are presented as the mean ± SD. *n* = 6 mice per group, ****p < 0.0001 vs. CUMS group.

The pro‐inflammatory factors such as TNF‐α, IL‐1β, and IL‐6 are classical markers of inflammation frequently studied in the context of depression. These cytokines play significant roles in mediating inflammatory responses, which have been linked to the development and progression of depressive symptoms. To investigate the impact of AE on inflammation, we measured the levels of TNF‐α, IL‐1β, and IL‐6 in both serum and brain tissue of mice. Our analysis revealed that AE treatment effectively reduced the concentrations of these pro‐inflammatory cytokines in serum compared to the CUMS group. In serum, compared to the control, levels increased in CUMS mice; compared to the CUMS group, they could be downregulated in the AE group (Figure 4A–C). The analysis of inflammatory factor content in the brain tissues of mice demonstrated that AE significantly lowered the levels of TNF‐α, IL‐1β, and IL‐6. Specifically, compared to the control group, the brain tissue of CUMS‐exposed mice showed a marked increase in TNF‐α, IL‐1β, and IL‐6 levels. However, when compared to the CUMS group, the AE‐treated group exhibited a significant reduction in these pro‐inflammatory markers (Figure 4D–F). These findings indicated that AE possesses antidepressant properties by effectively reducing the expression of inflammatory factors.

### 3.5 Effects of AE on CORT‐Induced Neurotoxicity

**FIGURE 5:**
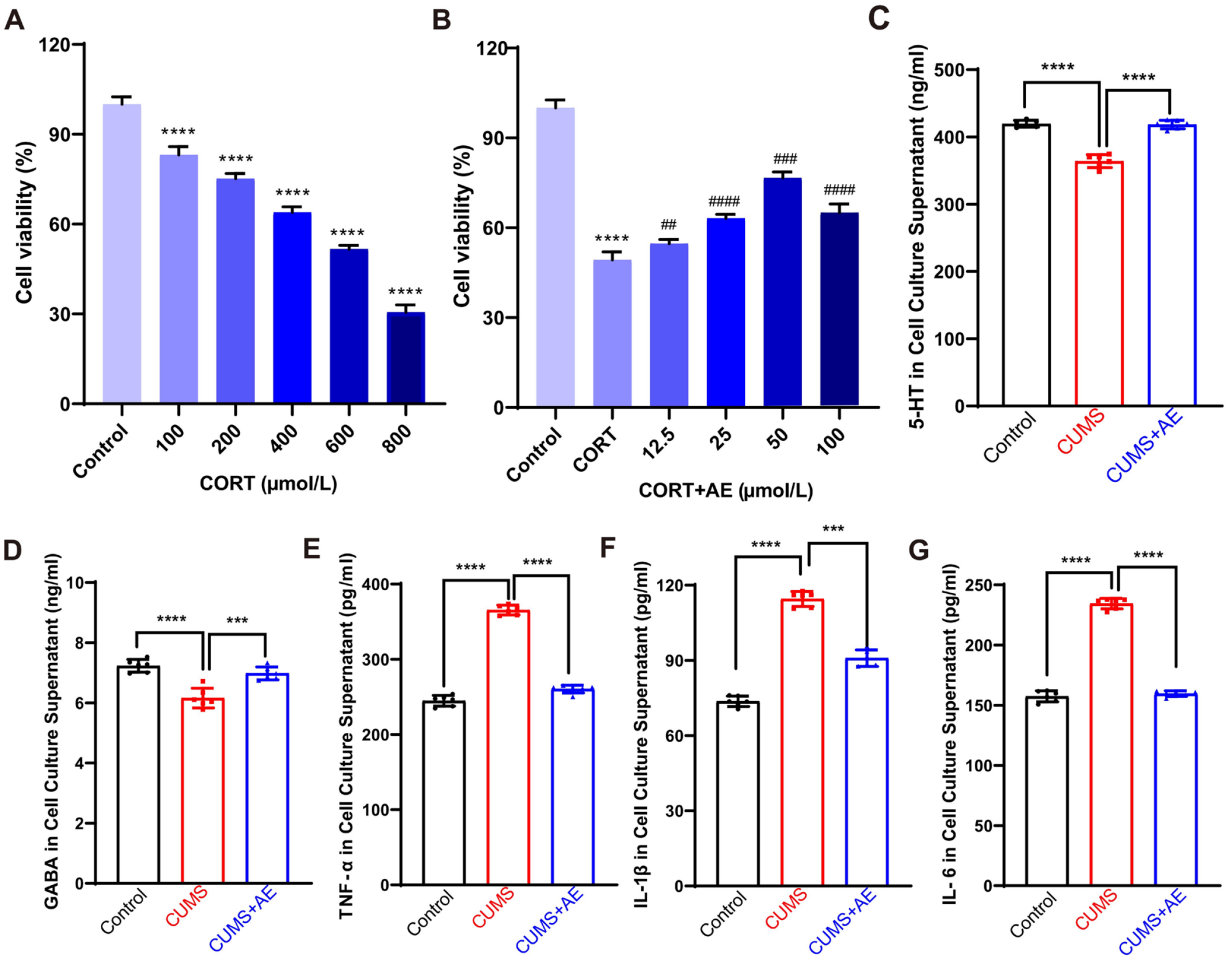
AE exerted significant neuroprotective effects against CORT‐induced cytotoxicity. To determine the protective effect of AE on CORT‐induced cytotoxicity in N2a cells, we measured the cell viability at various AE concentrations. (A) N2a cells were exposed to CORT at different concentrations (0, 100, 200, 400, 600 and 800 μmol/L) for 24 h and then were determined by CCK‐8 assay. (B) N2a cells were exposed to different concentrations of AE (12.5, 25, 50, 100 μmol/L) and 600 μmol/L CORT for 24 h. Quantification of 5‐HT (C), GABA (D), TNF‐α (E), IL‐1β (F), and IL‐6 (G) in cell culture supernatant harvested from the 600 μmol/L CORT‐induced depression‐like cell model intervened with AE (50 μmol/L). All data were presented as the mean ± SD. *n* = 6 cell culture supernatants per group, ^##^ *p* < 0.01, ^###^ *p* < 0.001, ^####^ *p* < 0.0001 vs. control group, ***p < 0.001, ****p < 0.0001 vs. CORT‐induced depression‐like cell model group.

To determine the neuroprotective effects of AE against cell cytotoxicity induced by high‐concentration CORT, we established an in vitro model mimicking stress‐induced depression. This study found that CORT at concentrations of 200, 400, 600, and 800 μmol/L led to a significant reduction in N2a cell viability. Specifically, exposure to 600 μmol/L CORT decreased cell viability to about 50%, making it the concentration chosen for further experiments (Figure 5A). Subsequently, we examined the cytotoxicity and protective effects of different AE concentrations. Intriguingly, AE treatments at 12.5, 25, 50, and 100 μmol/L notably reduced CORT‐induced cytotoxicity in a dose‐dependent manner (Figure 5B). Based on these results, we opted for 50 μmol/L AE in subsequent experiments involving the 600 μmol/L CORT‐induced depression cell model. In this model, compared to the control cells, the levels of 5‐HT and GABA were significantly lower, while TNF‐α, IL‐1β, and IL‐6 levels were markedly higher in the CORT‐induced depression cell model. These alterations mirrored those observed in CUMS‐induced depressed mice. Importantly, AE treatment reversed these changes, restoring the levels of 5‐HT and GABA and decreasing the levels of TNF‐α, IL‐1β, and IL‐6 (Figure 5C–G). The above results indicated that AE provides substantial neuroprotection against CORT‐induced cytotoxicity in vitro. By elevating key neurotransmitter levels such as 5‐HT and GABA and lowering pro‐inflammatory cytokines like TNF‐α, IL‐1β, and IL‐6, AE shows promise as a therapeutic agent for mitigating neuronal damage caused by chronic stress.

### 3.6 Effects of AE on cAMP/PKA/CREB/PTGS2 Signaling Pathway

**FIGURE 6:**
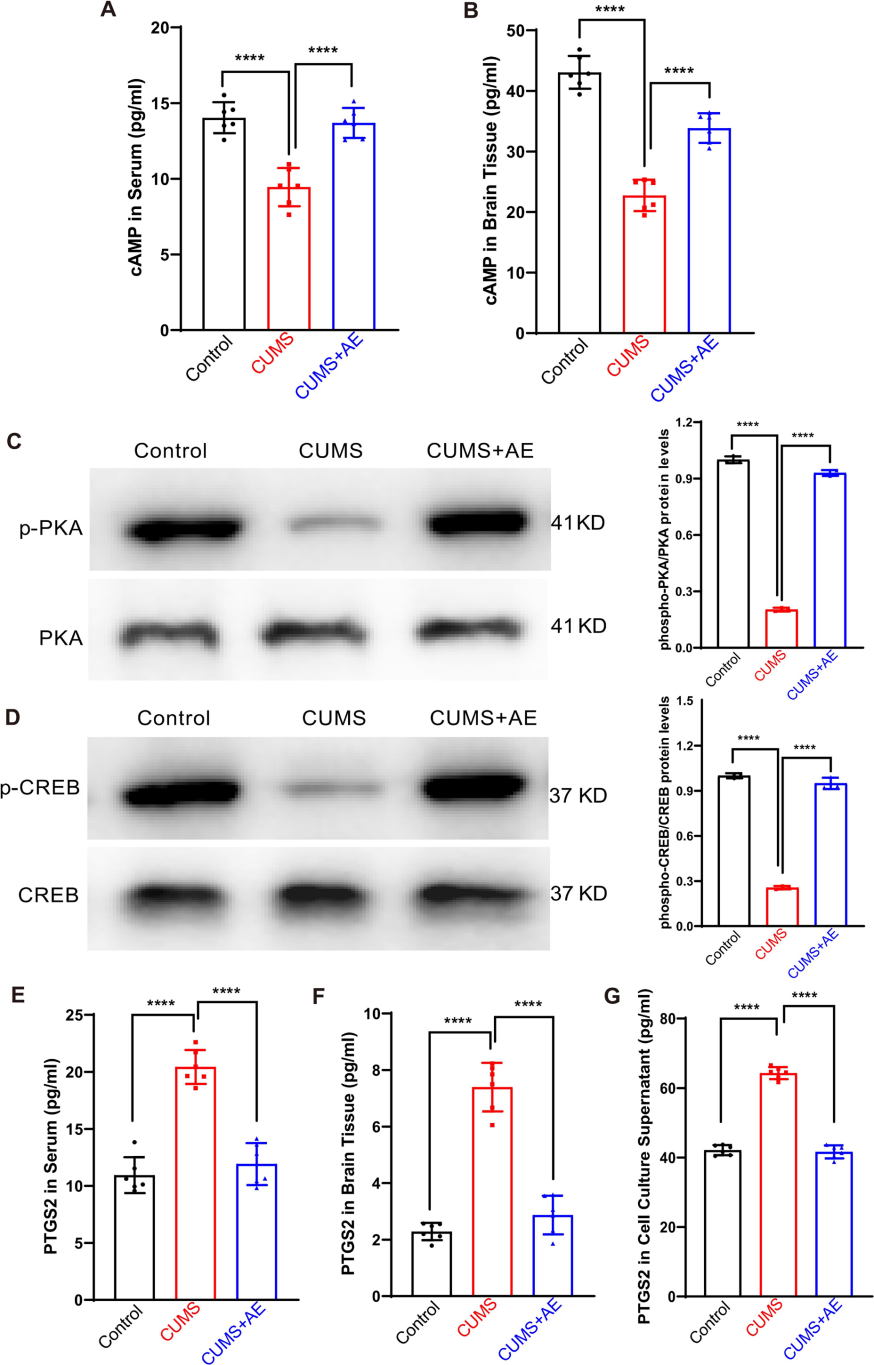
AE acts on the cAMP/PKA/CREB signaling pathway to inhibit PTGS2. (A and B) The expression levels of cAMP and PTGS2 were detected by ELISA in serum (A) and brain tissue (B). (C) Western blot bands: Band 1, control group; Band 2, CUMS group; Band 3, AE (50 μmol/L) + CUMS group. Phosphorylated PKA (pPKA) levels were normalized to total PKA levels. (D) Western blot bands: Band 1, control group; Band 2, CUMS group; Band 3, AE (50 μmol/L) + CUMS group. Phosphorylated CREB (pCREB) levels were normalized to total CREB levels. (E–G) Levels of PTGS2 in serum (E), brain tissue (F) and cell culture supernatant (G). All data were presented as the mean ± SD. *n* = 6 mice per group, ****p < 0.0001 vs. CUMS group.

To better understand the molecular mechanisms responsible for the protective effects of AE in CUMS mice, we utilized enzyme‐linked immunosorbent assay (ELISA) to quantify the levels of cyclic AMP (cAMP) in both serum and brain tissue. At the same time, Western blot was used to detect the levels of phosphorylated PKA (pPKA) and phosphorylated CREB (pCREB) in brain tissue. Moreover, ELISA was used to detect the levels of cAMP and PTGS2 in serum and brain tissue. Our results showed that cAMP, pPKA, and pCREB levels were suppressed in CUMS mice, but the expression level of PTGS2 was significantly higher compared to the control group. In the CUMS+AE group, coupled with the elevation of cAMP, pPKA, and pCREB levels, AE treatment significantly reduced PTGS2 level compared to the CUMS group (Figure 6A–F). Interestingly, compared to the control cells, the level of PTGS2 was elevated in the CORT‐induced depression cell model. These results were consistent with those observed in CUMS‐induced depressed mice and were reversed by AE treatment (Figure 6G). Our research indicated that the cAMP/PKA/CREB/PTGS2 signaling pathway may play a crucial role in the antidepressant‐like effects of AE.

## 4 Discussion

Depression, as one of the major diseases threatening human health, has garnered increasing attention and concern. Western medical treatments for depression are based on theories such as the monoamine neurotransmitter hypothesis and receptor hypotheses, but their mechanisms of action remain unclear, and they are often associated with a slow onset of efficacy and numerous adverse reactions. Therefore, screening effective alternative strategies is urgent. Studies have proved the obvious antidepressant effects of dietary polyphenols such as resveratrol, quercetin, and chlorogenic acid, particularly the effects on depression‐related inflammation (Tayab et al. 2022; Wang et al. 2022). Apple polyphenol extracts (APE), an active mixture rich in active monomers like chlorogenic acid, quercetin, and proanthocyanin B2, exhibit strong anti‐inflammatory, antioxidant, and antiaging biological activities (Li et al. 2019). Recent studies have shown that apple polyphenol extracts improve depressive‐like behaviors in high‐sugar diet‐fed mice by inhibiting inflammation in the gut‐brain axis (Xie, Wu, et al. 2024). Another study also demonstrated that apple polyphenol extracts alleviate depressive‐like behaviors in high‐sugar diet‐fed mice by modulating bile acid circulation through the liver‐gut‐brain axis mediated by the Farnesoid X Receptor (FXR) (Xie, Shang, et al. 2024). However, these studies have focused on the antidepressant effects of specific components in the apple, which may not fully reflect the interactions among multiple active components present in the apple. Therefore, this study seeks to uncover the antidepressant mechanisms of AE using a combination of network pharmacology analysis and traditional pharmacological techniques.

In this study, we identified 17 active components of AE through database screening. Subsequently, we retrieved 389 targets linked to AE and 1341 targets connected to depression. Among these, 118 common targets shared by both AE and depression were selected for subsequent analysis. PPI network analysis identified AKT1, TNF, ESR1, CASP3, PTGS2, and EGFR as the core targets of AE for antidepressant treatment. GO analysis of the 118 candidate targets indicated a notable enrichment in biological processes such as the response to xenobiotic stimulus and the inflammatory response. Increasing evidence has supported the antidepressant effects of the individual components of AE (e.g., chlorogenic acid and epicatechin) in a mouse model of depression (Martínez‐Damas et al. 2021; Song et al. 2019). Additionally, procyanidin B2 has been proven to decrease blood–brain barrier (BBB) leakage and restore the decreased expression of a tight junction protein, zonula occludens‐1 (ZO‐1), in both the cortex and striatum to attenuate BBB disruption in ischemic rats, suggesting that procyanidin B2 might also have an antidepressant effect (Wu et al. 2015). APE has been reported to effectively ameliorate sugary‐diet‐induced depression‐like behaviors in CUMS mice by reducing inflammation within the gut‐brain axis (Xie, Wu, et al. 2024). KEGG pathway analysis indicated that the candidate targets are significantly enriched in pathways like the cAMP signaling pathway. Growing evidence suggested that these pathways play a crucial role in the development and progression of depression (Gao et al. 2022; Li et al. 2009). These insights suggested that AE may effectively alleviate depression primarily by modulating the cAMP signaling pathway. This hypothesis highlights the necessity for further research to clarify the precise roles of these pathways in the antidepressant mechanisms of AE. AE contains a diverse array of compounds, each with multiple targets and pharmacological effects relevant to depression. This multifaceted composition may lead to synergistic effects, potentially reducing the side effects commonly associated with single‐action antidepressants. Our findings indicated that AE improves depressive‐like behavior in CUMS mice through several mechanisms, including the regulation of synaptic plasticity, enhancement of synaptic levels of monoamines, and inhibition of inflammatory factor expression. The CORT‐induced nerve injury cell model, which can simulate the neurotransmitter deficits, oxidative stress, inflammatory response, and neuronal loss associated with depression (Pan et al. 2023; Wang, Wang, Wang, et al. 2024; Wang, Hu, Liu, et al. 2024), is suitable for research on the effective mechanisms of drug relating to neurotransmitter imbalance or inflammatory cytokine changes in vitro. Our results demonstrated that 50 μmol/L AE was the optimal concentration for treating N2a cell injury induced by 600 μmol/L CORT in vitro. Furthermore, the protective effects of AE were associated with the promotion of neurotransmitter synthesis and inhibition of inflammatory reactions. In our study, AE has demonstrated therapeutic efficacy in both in vivo and in vitro depression models by correcting imbalances in neurotransmitters and inflammation. This dual‐action approach highlights AE's potential as a comprehensive treatment for depression, addressing multiple underlying mechanisms simultaneously.

Intracellular signal transduction pathways are currently a focus and challenge in the research of antidepressant mechanisms. Cyclic adenosine monophosphate (cAMP) is a crucial second messenger within cells, mediating the actions of various intracellular hormones, neurotransmitters, and other signaling molecules. Adenylyl cyclase (AC) is a key enzyme in G protein‐coupled signaling pathways, converting adenosine triphosphate (ATP) into the second messenger cAMP. cAMP then activates protein kinase A (PKA), which in turn phosphorylates various proteins, including transcription factors, ion channels, cytoskeletal elements, and enzymes. One of these phosphorylated transcription factors is the cAMP response element‐binding protein (CREB). CREB is a nuclear protein composed of 341 amino acids. It plays a critical role in neuronal development, regeneration, synapse formation, and cellular repair, making it one of the key factors in regulating central nervous system function (Dinevska et al. 2024). It is an important molecular target in the treatment of depression. Recent studies have suggested that alterations in hippocampal CREB are closely related to the pathogenesis of depression and the effectiveness of antidepressant therapy. The expression and activation of hippocampal CREB are influenced by various signaling molecules and pathways. Multiple signaling pathways activated by antidepressants ultimately lead to the phosphorylation of the nuclear transcription factor CREB, thereby regulating the expression of many genes that promote cell growth, proliferation, survival, and neural plasticity, such as brain‐derived neurotrophic factor (BDNF), nerve growth factor (NGF), vascular endothelial growth factor (VEGF), insulin‐like growth factor I (IGF‐I), and activin, thus exerting antidepressant effects (McCauslin et al. 2006). This indicates that CREB is a key protein for the efficacy of many antidepressant drugs and can be activated by various signaling pathways. As a transcription factor, CREB participates in the transition from short‐term to long‐term memory, and its phosphorylation is regulated by multiple signaling pathways, including the classic AC/cAMP/PKA pathway (Dowlatshahi et al. 1998). The AC/cAMP/PKA pathway plays a significant role in mood regulation and is one of the earliest and most extensively studied signal transduction pathways in antidepressant research. The general process of the cAMP signaling cascade is as follows: exogenous stimuli cause the release of neurotransmitters (such as serotonin), which bind to corresponding receptors on the cell membrane (such as G protein‐coupled receptors). G proteins can regulate the activity of AC, catalyzing the conversion of ATP to cAMP, which in turn activates PKA, leading to the phosphorylation of CREB. The phosphorylation of CREB enables the induction and regulation of related genes (Shaywitz and Greenberg 1999). Studies have found that antidepressants can activate G protein coupling in blood cells of depressed patients, specifically increasing the coupling of Gs protein with adenylyl cyclase (Gs‐AC) (Grønli et al. 2004). cAMP is currently recognized as one of the key signaling pathways associated with antidepressant effects. Under normal physiological conditions, cAMP is synthesized from ATP through the enzymatic action of AC and is mainly degraded by type IV phosphodiesterase (PDE4). The functional states of AC and PDE4 together maintain the stability of cAMP levels in neurons. Branski et al. discovered that the PKA agonist 8‐Br‐cAMP could dose‐dependently decrease the immobility time of rats in the forced swim test, indicating its antidepressant properties. This study provided the first animal evidence of the antidepressant action of a PKA agonist (Brański et al. 2008). Li et al. used the specific PDE4 inhibitor rolipram to inhibit the hydrolysis of cAMP in mice. After continuous administration for 17–23 days, the levels of cAMP and pCREB in the hippocampus were upregulated, reducing the immobility time in the forced swim and tail suspension tests, similar to the behavioral changes observed after fluoxetine administration. This suggests that the activation of the cAMP pathway in the hippocampus plays a positive role in improving depressive symptoms (Li et al. 2009), consistent with Branski's findings. Clinical studies have confirmed that the levels of cAMP and PKA activity in the prefrontal cortex of suicide victims with depression are reduced, along with the expression of neuroprotective and synaptic plasticity molecules activated by cAMP or PKA, such as Rap‐1 (Dwivedi et al. 2006). Another clinical study found that PKA activity is decreased in peripheral blood and post‐mortem brain tissue samples from depressed patients, particularly in those with severe depression and suicidal tendencies (Shelton et al. 2009). Many studies have indicated that the cAMP signaling pathway, through intracellular PKA, plays a crucial role in antidepressant therapy (Gao et al. 2022). Chang Shen Hua volatile oil (CSHVO) improves CORT‐induced injury in the PC12 cell model and alleviates depression‐like behaviors in a CUMS combined with orphan rearing model in rats. The antidepressant mechanism of CSHVO is associated with the modulation of the cAMP‐PKA‐CREB signaling pathway (Zhang et al. 2024). These research findings suggested that the cAMP signaling pathway is downregulated in depressed patients, and antidepressant treatment can upregulate the function of the cAMP pathway. In our study, compared to the control group, the expression of cAMP, PKA, and CREB in the brain tissue of the depression model mice was significantly decreased, consistent with previous literature reports. After 5 weeks of intraperitoneal administration of AE, the protein expression of cAMP, PKA, and CREB was significantly increased, indicating that the mechanism of AE's antidepressant effect is related to the cAMP/PKA/CREB signaling pathway.

PTGS2 (Prostaglandin‐Endoperoxide Synthase 2, also known as COX‐2) is an inducible enzyme that plays a crucial role in the inflammatory response. There is a close association between PTGS2 and depression, involving multiple aspects, including neuroinflammation, neurotransmitter metabolism, neuroplasticity, and hypothalamic–pituitary–adrenal (HPA) axis function (He et al. 2022). Previous studies have demonstrated that COX‐2 and PGE2 are upregulated in a rat model of depression, and inhibition of the COX‐2/PGE2 pathway can mitigate the inflammatory response in the hippocampus and effectively alleviate depressive behaviors (Song et al. 2018). Studies have shown that kaempferol exerts antidepressant effects, at least partially through the inhibition of the COX‐2/PGE2 pathway, which modulates neuroinflammation, neurotransmitter imbalance, and impaired neurogenesis (Zhu et al. 2023). Another study found that bergapten alleviates depression‐like behavior by inhibiting COX‐2 activity and the NF‐κB/MAPK signaling pathway in microglia (Yan et al. 2023). Additionally, a recent study also found that quercetin inhibits neuronal ferroptosis and enhances immune responses in breast cancer‐related depression (BCRD) mice by targeting the lipid metabolism‐related gene PTGS2 (Zhu et al. 2024). In our study, it was found that the relative protein expression of PTGS2 in the model group was markedly elevated compared to the normal control group, aligning with earlier research findings. This increase in PTGS2 levels underscores its potential role in the pathophysiology of depression. After 5 weeks of AE treatment, the relative protein expression of PTGS2 in the treated group was significantly reduced compared to the model group, indicating a substantial impact of AE on this inflammatory marker. Thus, our study suggested that alongside the improvement of cognitive functions and depression‐like behaviors, AE treatment showed a regulatory effect on the cAMP/PKA/CREB/PTGS2 signaling pathway in CUMS mice.

## 5 Conclusions

In summary, our study demonstrated that cAMP/PKA/CREB/PTGS2 is one of the potential signaling pathways for depression treatment. Through targeting the cAMP/PKA/CREB/PTGS2 signaling pathway, AE can regulate synaptic plasticity and neurotransmitters, inhibit inflammatory responses, and reduce neuronal toxicity, thereby improving cognitive dysfunction and alleviating depressive‐like behavior in CUMS mice. These findings highlighted the potential of AE as a promising treatment option for depression. The regulatory effect of AE on the cAMP/PKA/CREB/PTGS2 signaling pathway suggested its ability to address multiple aspects of depression. However, further research is essential to fully elucidate the specific mechanisms involved and to optimize dosage and administration strategies for more effective utilization of AE in depression therapy.

## Author Contributions

**Zhu Li:** conceptualization (lead), data curation (equal), formal analysis (equal), funding acquisition (lead), investigation (equal), methodology (equal), project administration (lead), visualization (equal), writing – original draft (equal), writing – review and editing (lead). **Yue Chen:** conceptualization (equal), data curation (equal), formal analysis (equal), investigation (equal), methodology (equal), writing – original draft (equal). **Shunqiang Yang:** conceptualization (equal), data curation (equal), investigation (equal), writing – review and editing (equal). **Huihuang Shi:** investigation (equal), visualization (equal), writing – original draft (equal). **Jizhe Cui:** visualization (equal), writing – original draft (supporting).

## Conflicts of Interest

The authors declare no conflicts of interest.

## Data Availability

The data that support the findings of this study are available from the corresponding author upon reasonable request.
